# Determination of 19 Steroid Hormones in Human Serum and Urine Using Liquid Chromatography-Tandem Mass Spectrometry

**DOI:** 10.3390/toxics10110687

**Published:** 2022-11-12

**Authors:** Zhong-Min Li, Kurunthachalam Kannan

**Affiliations:** 1 Department of Pediatrics, New York University Grossman School of Medicine, New York, NY 10016, USA; 2 Department of Environmental Medicine, New York University Grossman School of Medicine, New York, NY 10016, USA

**Keywords:** steroid hormones, estrogens, androgens, progestogens, corticosteroids, urine, serum

## Abstract

This paper describes a methodology for simultaneous determination of 19 steroid hormones, *viz*. estrone, estradiol, estriol, testosterone, 5α-dihydrotestosterone, androstenedione, androstenediol, dehydroepiandrosterone, progesterone, pregnenolone, 17α-OH-progesterone, 17α-OH-pregnenolone, cortisone, cortisol, 11-deoxycortisol, 11-deoxycorticosterone, 11-dehydrocorticosterone, aldosterone, and corticosterone, in 500-µL of urine or serum/plasma. The method was optimized using isotopically labeled internal standards and liquid-liquid extraction followed by detection using liquid chromatography-electrospray ionization-tandem mass spectrometry (LC-MS/MS). Dansylation of estrogens significantly improved their sensitivities (~11- to 23-fold) and chromatographic separation. The respective limit of detection (LOD) and limit of quantification (LOQ) of all analytes were 0.04–0.28 and 0.14–0.92 ng/mL in human urine, and 0.11–0.35 and 0.38–1.18 ng/mL in human serum/plasma. Recoveries of all analytes (except for progesterone) fortified at 10, 20, and 200 ng/mL in urine and serum were 80–120%, with standard deviations ranging from 0 to 17.3%. Repeated analysis of similarly fortified urine and serum samples yielded intra-day and inter-day variations of 0–21.7% and 0.16–11.5%, respectively. All analytes except cortisone exhibited weak matrix effects in urine and serum (−13.9–18.2%). The method was further validated through the analysis of the National Institute of Standards and Technology (NIST) plasma Standard Reference Material (SRM1950) with certified concentrations for cortisol, progesterone, and testosterone (coefficient of variation: 3–11%). The developed method was applied in the analysis of urine samples from 20 volunteers, which revealed the occurrence of 16 analytes with detection frequencies (DFs) > 80%. Furthermore, 15 analytes were found in plasma SRM1950, indicating the feasibility of our method in the analysis of steroid hormones in urine and serum/plasma. This method will facilitate analysis of steroid hormones in population-based biomonitoring studies.

## 1. Introduction

**Figure 1:**
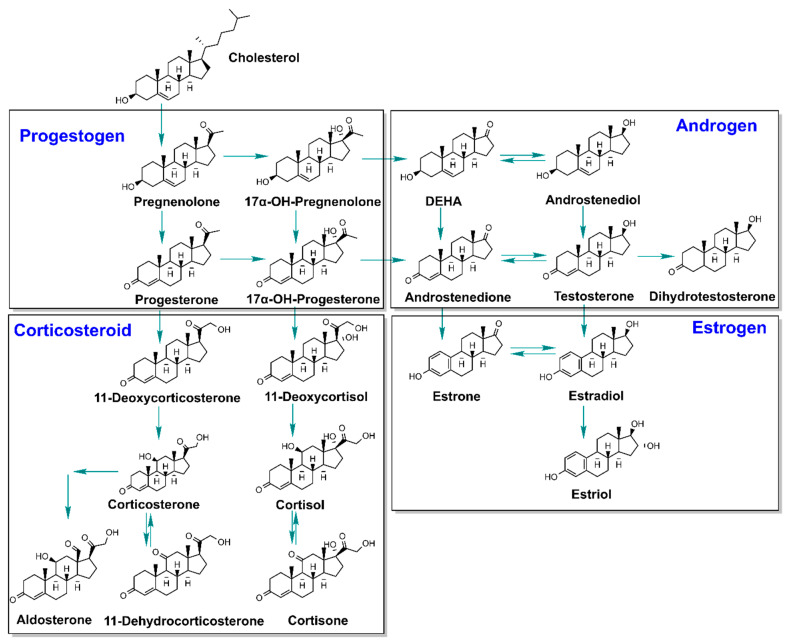
Molecular structures and metabolic pathway of the 19 steroid hormones investigated in this study.

Steroid hormones are cholesterol derivatives that play critical roles in regulating water and salt homeostasis, metabolism, stress response, and in initiating and maintaining sexual differentiation and reproduction [1]. Steroid hormones are synthesized through a cascade-like pathway in the adrenal cortex, the gonads and the placenta and are released into the blood stream to act in both peripheral target tissues and the central nervous system. The homeostasis of steroid hormones is regulated through hypothalamus-pituitary-gonadal (HPG) and hypothalamus-pituitary-adrenal (HPA) axes [2,3], which consist stimulatory hormones and feedback loops. Steroid hormones can be classified as estrogens, androgens, progestogens, and corticosteroids (Figure 1), according to their structures and genomic receptors to which they bind [4]. In general, estrogens (18 carbons with an aromatic ring) are female reproductive hormones, androgens (19 carbons) are male reproductive hormones, progestogens (21 carbons) are pregnancy hormones, and corticosteroids (21 carbons) are stress hormones [5].

The lipophilic steroid hormones undergo phase I and phase II metabolism, and are excreted mainly through urine as glucuronides, sulfates, diglucuronides, disulfates, and sulfoglucuronides [1,6]. Perturbation in steroid hormone homeostasis in blood, urine, saliva, and hair has been measured in investigations focused on cancer [7,8,9,10,11], stress [12], and endocrine-disruption by environmental chemicals such as bisphenols [13], phthalates [14] and triclosan [13].

Traditional methods of analysis of steroid hormones in human specimens (such as serum) are radioimmunoassays (RIAs) and enzyme-linked immunosorbent assays (ELISAs). These methods are highly sensitive, but lack selectivity due to nonspecific antigen-antibody interactions [15]. Besides, RIAs and ELISAs usually target one analyte (i.e., hormone) per assay. Methods based on mass spectrometry offer improved robustness, specificity and accuracy, and have been routinely used for steroid hormone analysis in recent years. For instance, gas chromatography-mass spectrometry (GC-MS) methods provide high sensitivity, selectivity and accuracy. However, due to the low volatilities of steroid hormones, analysis by GC-MS requires derivatization, a laborious and time consuming step [16,17]. To overcome this problem, studies have applied liquid chromatography coupled with tandem mass spectrometry (LC-MS/MS) [18,19,20,21,22]. Nevertheless, challenges exist in simultaneous measurement of ultra-trace levels of several steroid hormones in serum or urine. Estrogens exhibit low sensitivities due to their low ionization efficiencies [23], leading to difficulties in analysis at trace levels of these hormones [24]. Besides, many steroid hormones have similar chemical structures and molecular fragmentation patterns, which make their mass spectrometric identification difficult. The loss of one or two water moiety from the molecule is a common mass fragmentation feature observed for steroid hormones. Several reported earlier studies entailed long time (e.g., 40 min) for chromatographic separation [5,19], which can hamper high throughput analysis in large-scale population-based studies.

We describe a method for sensitive and selective determination of four classes of steroid hormones simultaneously in human urine and serum/plasma. The goals of this study were: (1) to develop a LC-MS/MS method for the determination of physiologically relevant concentrations of 19 steroid hormones in a single extraction and injection; (2) to evaluate and validate the method for sensitivity, accuracy and precision in human urine and serum/plasma; and (3) to assess the feasibility of the method in measuring steroid hormones in urine samples from the general population and in pooled human serum/plasma. Nineteen steroid hormones, comprising 3 estrogens (estrone, estradiol and estriol), 5 androgens (testosterone, 5α-dihydrotestosterone, androstenedione, androstenediol, and dehydroepiandrosterone (DEHA)), 4 progestogens (progesterone, pregnenolone, 17α-OH-progesterone, and 17α-OH-pregnenolone), and 7 corticosteroids (cortisone, cortisol, 11-deoxycortisol, aldosterone, 11-deoxycorticosterone, 11-dehydrocorticosterone, and corticosterone) were the target analytes.

## 2. Materials and Methods

### 2.1. Chemicals and Reagents

The chemical structures of the 19 steroid hormones investigated in this study are shown in Figure 1. Individual certified stock solutions of cortisone, 11-deoxycortisol, aldosterone, corticosterone, progesterone, 17α-OH-progesterone, pregnenolone, estrone, estradiol, testosterone, dehydroepiandrosterone (DEHA), 5α-dihydrotestosterone, 17α-OH-pregnenolone, 11-dehydrocorticosterone, androstenediol, ^13^C_3_-cortisone, ^13^C_3_-cortisol, ^13^C_3_-11-deoxycortisol, D_4_-aldosterone, D_4_-corticosterone, ^13^C_3_-progesterone, ^13^C_3_-17α-OH-progesterone, ^13^C_2_-D_2_-pregnenolone, ^13^C_3_-estrone, ^13^C_2_-estradiol, ^13^C_3_-estriol, ^13^C_3_-testosterone, ^13^C_3_-DEHA, ^13^C_3_-5α-OH-dihydrotestosterone, and ^13^C_2_-D_2_-17α-OH-pregnenolone (100 µg/mL) with purities of 95–98% were purchased from Cambridge Isotope Laboratories (Andover, MA, USA). Neat cortisol and estriol (purity: ≥95%) were obtained from Cambridge Isotope Laboratories (Andover, MA, USA). Individual stock solutions of cortisol and estriol (100 µg/mL) were prepared in methanol (MeOH). Individual certified stock solutions of 11-deoxycorticosterone, androstenedione and ^13^C_3_-11-deoxycorticosterone (100 µg/mL) with purities of ≥98% were purchased from Sigma-Aldrich (St. Louis, MO, USA). All stock solutions were stored at −20 °C. Working solutions of analytical standards were diluted from stock solutions using acetonitrile (ACN).

Synthetic urine was purchased from Cerilliant (Round Rock, TX, USA). Pooled human serum was from Sigma-Aldrich (St. Louis, MO, USA). Plasma Standard Reference Material (SRM1950) was purchased from the National Institute for Standards and Technology (NIST; Gaithersburg, MD, USA) through Sigma-Aldrich (St. Louis, MO, USA). β-glucuronidase/arylsulfatase enzyme (ALS; β-glucuronidase: ~100,000 units/mL; arylsulfatase: ~47,500 units/mL) from *Helix pomatia* was obtained from Roche Life Science through Sigma-Aldrich (St. Louis, MO, USA). _L_-Ascorbic acid, dansyl chloride (DC), formic acid (88%), and ammonium acetate (NH_4_Ac) of analytical grade were from Sigma-Aldrich (St. Louis, MO, USA). Water, ACN, methyl *tert*-butyl ether (MTBE), and ethyl acetate (EtAc) of LC-MS grade were from Fisher Scientific (Waltham, MA, USA). Bond Elut C18 (60 mg/3 mL), Bond Elut Plexa (60 mg/3 mL), and Bond Elut NEXUS (60 mg/3 mL) solid phase extraction (SPE) cartridges were obtained from Agilent Technologies (Santa Clara, CA, USA).

### 2.2. Sample Collection

Spot urine samples were collected randomly in 50-mL polypropylene (PP) tubes from 11 male (age: 18–64 years, average 38.0 years) and 9 female (age: 15–59 years, average 33.3 years) volunteers from New York City during May–June 2022. Immediately after collection, samples were stored at −20 °C until analysis. The urine samples were deidentified and therefore fell under the ‘exempt’ category of the New York University Institutional Review Board.

### 2.3. Preparation of Urine and Serum Samples

A 500-µL aliquot of urine was pipetted into a 15-mL borosilicate glass culture tube. Synthetic urine and pooled human urine fortified with target analytes (at concentrations of 10, 20, and 200 ng/mL) were used for method development and validation. Then, 2.5–12.5 ng of isotopically labelled internal standards were spiked into each sample, and 500 µL of 1 M NH_4_Ac buffer (pH 5.5) containing 2.5 mg of _L_-ascorbic acid and 20 µL of ALS enzyme (2000 units) were added. After gentle mixing, the samples were incubated overnight (~15 h) at 37 °C by shaking at 100 rpm (Jeio Tech Co., Seoul, South Korea). Thereafter, 1 mL of HPLC-grade water was added to each sample, followed by the addition of 4 mL MTBE/EtAc (5:1, *v/v*). After ultra-sonication for 15 min and shaking for 30 min in a reciprocating shaker, the samples were subjected to centrifugation at 3000 rpm for 5 min, and the supernatant was transferred into a new glass tube. The liquid-liquid extraction (LLE) was repeated twice with 3 mL of MTBE/EtAc (5:1, *v/v*). The combined extracts were subsequently evaporated to dryness under N_2_ at 25 °C. Estrogens were then selectively derivatized by the addition of 125 µL of sodium bicarbonate buffer (0.1 M; pH 9.0) and 125 µL of dansyl chloride (1 mg/mL in acetone), vortexed vigorously (~30 s), and the sample tubes were immediately kept at 60 °C (on a hot plate) for 5 min. The extracts were evaporated to dryness under N_2_ at 25 °C, reconstituted in 250 µL MeOH, and transferred into amber glass vials for LC-MS/MS analysis.

Serum samples (500 µL) were processed by following the procedure described above, without enzymatic hydrolysis. Pooled human serum fortified with target analytes (at concentrations of 10, 20, and 200 ng/mL) was used for method development and validation.

### 2.4. LC-MS/MS Analysis

The quantification of target analytes was performed on an ABSciex 5500+ Q-trap mass spectrometer (Framingham, MA, USA) coupled to an ExionLC HPLC (SCIEX, Redwood City, CA, USA). Analytes were separated on an Eclipse Plus C18 RRHD column (150 × 2.1 mm, 1.8 µm; Agilent Technologies, Santa Clara, CA, USA) coupled to a BetaSil C18 guard column (20 × 2.1 mm, 5 µm; Thermo Fisher Scientific, Waltham, MA, USA). The mobile phase, maintained at a flow rate of 0.2 mL/min, consisted water (A) and ACN (B) each containing 0.1% formic acid (*v/v*). The following gradient program was applied: hold at 10% B for 0.5 min, linear ramp to 40% B over 0.5 min, linear ramp to 70% B over 9 min, then linear ramp to 95% B over 0.5 min, hold at 95% B for 4.5 min, then return to initial conditions over 0.5 min, and equilibrate for 1.5 min prior to the next injection. The mobile phase flow was diverted to waste during the first 3.5 min after sample injection. The column was maintained at room temperature; the autosampler was kept at 15 °C; and the injection volume was 10 µL.

All analytes were measured using electrospray ionization (ESI) multiple reaction monitoring (MRM) in positive-ion mode. The optimized MRM parameters including collision energy (CE), declustering potential (DP), collision cell exit potential (CXP), entrance potential (EP), and dwell time are given in Table 1 and Table S1 (Supplementary Material). The IonSpray voltage was 5.5 kV; the ion source temperature was 500 °C; the curtain gas and collision gas flow rates were set at 20 and 9 psi, respectively; and the ion source gases 1 and 2 were set at 70 and 60 psi, respectively. Analyst software v1.7.2 (ABSciex, Framingham, MA, USA) was used for data analysis.

### 2.5. Method Validation

Calibration curves of all analytes were constructed both in solvent and in fortified urine and serum matrices. Calibration standards were prepared in ACN at concentrations ranging from 0.05 to 2000 ng/mL, with 10–50 ng/mL of isotopically labelled internal standards, and were derivatized by following the procedure described above. The matrix-matched calibration curves were obtained by spiking all analytes at different concentrations (0.05–2000 ng/mL) in urine and serum extracts.

Matrix effects were evaluated by calculating the percentage of signal enhancement or suppression, as shown in Equation (1):

(1) $$\mathrm{Matrix}\;\mathrm{effect}=\left(\frac{A}{B}-1\right)\times 100\%$$

where A and B are the slopes of the matrix-matched calibration curve, and calibration curve from pure solvent, respectively.

Accuracy of the method was determined as the recoveries of analytes spiked at three different concentrations (10, 20 and 200 ng/mL) in both pooled urine and serum. Precision was assessed as intra-day and inter-day variations in measured concentrations in three pooled urine or serum samples fortified at 10, 20 and 200 ng/mL and was calculated as the coefficient of variation (CV%). The inter-day CV was measured by repeated injection of fortified samples on three different days. Accuracy of the method was further assessed through the analysis of NIST SRM1950 plasma, which has certified reference values for cortisol, progesterone and testosterone.

To calculate the method limit of detection (LOD) and limit of quantification (LOQ) of steroid hormones in urine, six replicates of synthetic urine were fortified with each target analyte at 1 ng/mL, a concentration that yielded signal-to-noise (*S/N*) ratios of 6.4–62.4. LOD and LOQ of target analytes in urine were calculated as 3 and 10 times the standard deviation (SD), respectively. Similarly, LOD and LOQ in serum were calculated as 3 and 10 times the SD of concentrations, respectively, measured in pooled human serum spiked at 1 ng/mL.

### 2.6. Quality Assurance and Quality Control

Quality control (QC) samples include procedural blanks (LC-MS grade water instead of urine or serum), matrix blanks (pooled urine or serum), and matrix spiked samples (analytes fortified in pooled urine or serum at three different concentrations: 10, 20 and 200 ng/mL). The NIST SRM sample was also included in the analysis of hormones in plasma.

## 3. Results and Discussion

### 3.1. Chromatography and Mass Spectrometry

The development of the method started with optimization of MS/MS parameters for 19 target analytes, through the infusion of a standard solution (200 ng/mL in MeOH containing 0.2% formic acid) directly into the mass spectrometer. Addition of formic acid to the standard solution (as an additive) over acetic acid yielded better signal intensity. ACN, as the solvent, yielded lower backpressure in comparison to MeOH.

**Figure 2:**
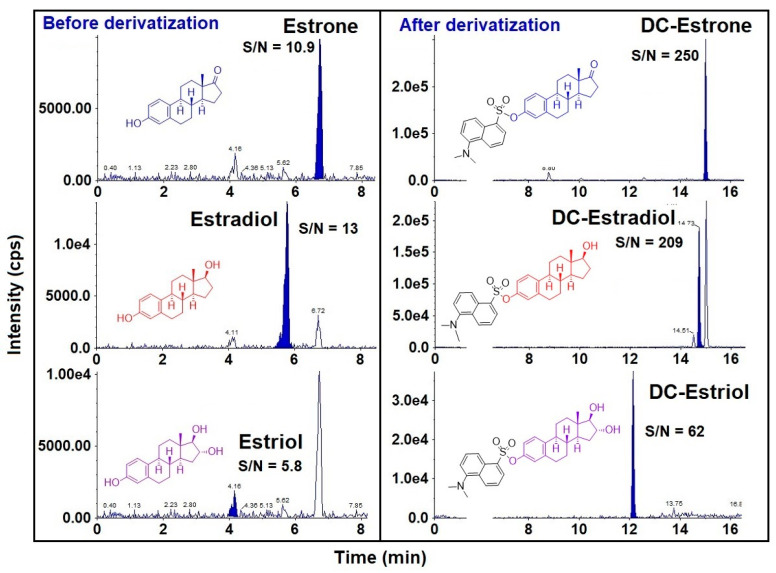
Comparison of LC-MS/MS responses of estrone, estradiol, and estriol before and after dansylation. Concentrations of the analytes were 1 ng/mL; Injection volume was 10 µL.

**Figure 3:**
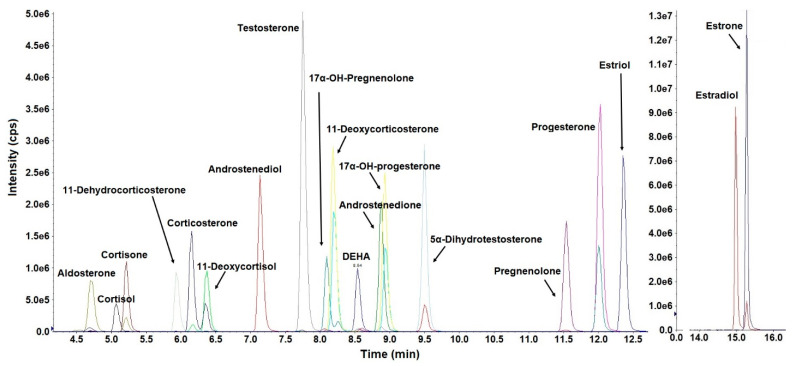
Representative LC-MS/MS chromatograms of 19 steroid hormones in solvent. Concentrations of the target analytes were 100 ng/mL; Injection volume was 10 µL; estrone, estradiol, and estriol were derivatized using dansyl chloride. Estrone and estradiol were shown separately because of their significantly higher intensities than the other analytes.

Chromatographic separation of steroid hormones in urine or serum is critical because several of these analytes exhibit the same mass spectrometric MRM transitions (Table 1). Few earlier studies employed lengthy chromatographic separation time to isolate and resolve individual analytes [5,19]. We compared the performance of several reversed-phase LC columns and found that Eclipse Plus C18 RRHD column (150 × 2.1 mm, 1.8 µm; Agilent Technologies, Santa Clara, CA, USA) provided good peak shape and chromatographic separation for most of the analytes within a run time of 10 min (Figure S1). Nevertheless, several important biomarkers were not resolved chromatographically. Estriol and aldosterone, as well as estrone and DEHA co-eluted at the same retention times. Furthermore, estrogens exhibited low signal intensities (Figure 2) due to their low ionization efficiencies. To improve selectivity and sensitivity of estrogens, we applied an estrogen-specific derivatization step using dansyl chloride, as reported earlier [18,19,25]. Following dansylation, the signal intensities of estrone, estradiol, and estriol improved significantly with an enhancement in *S/N* by 23, 16 and 11 times, respectively, in comparison to intensities obtained prior to derivatization. These results are consistent with those reported earlier [26]. Furthermore, the retention times of dansyl-estrone (DC-estrone), DC-estradiol, and DC-estriol were different from all other analytes, indicating improved selectivity (Figure 2 and Figure 3). Nevertheless, androstenedione and 17α-OH-progesterone co-eluted, which, however, was deemed acceptable because of their different MRM transitions (Table 1). Our method, with a total run time of 17 min, was faster than the previous methods [5,19], a feature favorable for large-scale human biomonitoring studies.

### 3.2. Optimization of Sample Purification

Given the lipophilic nature of steroid hormones, liquid-liquid extraction (LLE) with non-polar solvents (e.g., MTBE, EtAc and dichloromethane) and SPE with reversed-phase sorbents (e.g., C18) were considered appropriate to efficiently extract the analytes from urine and serum/plasma [19,20,26]. We compared the performance of LLE using MTBE/EtAc (5:1, *v/v*) [20] and different types of SPE cartridges including Bond Elut C18, Bond Elut Plexa, and Bond Elut NEXUS (Supplementary Material). The recoveries of most of the target analytes through the extraction (LLE) and purification (SPE) steps were acceptable (Table S2). However, strong matrix effect following SPE (Figure S2) resulted in poor resolution of pregnenolone and ^13^C_2_-D_2_-pregnenolone. Resolution of this compound was excellent in serum samples after LLE. Therefore, samples were analysed using LC-MS/MS after LLE with MTBE/EtAc (5:1, *v/v*).

In human urine, steroid hormones are present almost exclusively as phase II metabolites. Thus, deconjugation is an essential step to measure the total concentrations of steroid hormones. ALS enzyme was reported to be efficient for the deconjugation of phase II metabolites of various endogenous and exogenous compounds [20,27]. Therefore, urine samples were incubated with ALS enzyme prior to extraction.

### 3.3. Method Validation

A 16-point calibration curve was prepared at concentrations ranging from 0.05 to 2000 ng/mL, with 10–50 ng/mL of isotopically labelled internal standards. Calibration curves prepared in pure solvent yielded R-values in the range of 0.9973–0.9999 for all steroid hormones (Table S3). Matrix-matched calibration curves at fortification levels of 0.05–2000 ng/mL also exhibited excellent R-values in both synthetic urine (0.9974–0.9998) and pooled human serum (0.9958–0.9998) (Table 2 and Table 3).

Accuracy of the method was assessed by spike-recovery tests conducted in triplicate in pooled human urine and pooled human serum. Average recoveries of each analyte at different fortification levels (10, 20, and 200 ng/mL) were measured. In human urine, the recoveries of all hormones (except for progesterone) at three fortification levels were in the range of 81.7–117%, 85.0–120%, and 82.7–119%, respectively, and the relative standard deviation (SD) of triplicate measurements was 1.00–15.5%, 0–13.0%, and 0.30–11.0%, respectively. These results suggest acceptable accuracies for most of the analytes. Nevertheless, recoveries of progesterone were in the range of 117–141% (Table 2). This may indicate that certain untargeted hormone metabolites (or precursors) exhibit the same retention and MRM transition with progesterone, considering their similar chemical structures [1]. In human serum, the recoveries of all hormones at three fortification levels were in the range of 88.5–119%, 80.0–118%, and 88.7–119%, respectively, and the SD of triplicate analyses was in the range of 1.2–14.1%, 1–17.3%, and 1.0–8.8%, respectively (Table 3). The determined accuracies of all analytes were similar to those reported in an earlier study (84–122%) [20].

We further assessed and validated the accuracy of the method through the analysis of NIST human plasma SRM1950. The concentrations of testosterone, progesterone, and cortisol determined in our method agreed well with the certified reference values, with variations within 11% (Table 4), further confirming validity of the developed method.

We assessed precision of the method as intra-day and inter-day variations from repeated analysis of fortified human urine and serum samples. In human urine, the intra-day CVs of target analytes fortified at 10, 20, and 200 ng/mL were 0.60–19.0%, 0–11.2%, and 0.32–9.05%, respectively, whereas the inter-day CVs were 0.71–9.68%, 0.56–7.59%, and 0.55–4.77%, respectively (Table 2). In human serum, the intra-day CVs were 1.20–15.7%, 1.08–21.7%, and 0.64–9.32%, respectively, whereas the inter-day CVs were 0.16–11.5%, 0.78–8.55%, and 0.78–5.82%, respectively (Table 3). These results suggested acceptable precision (CVs < 20%) for all analytes except cortisol in serum (9.32–21.7%). The relatively low precision of cortisol was likely due to its high background concentration in urine and serum (Table 4 and Table S4). The method precision calculated in this study was similar to that reported in an earlier study (intra-day CV: 2–11%; inter-day CV: 5–24%) [20].

Matrix effect is a common phenomenon in ESI-LC-MS analysis, produced by ionization enhancement or suppression by matrix components. Typically, values in the range of −20 to +20% are considered as weak suppression/enhancement. Use of isotopically labelled internal standards of analytes (and isotopic dilution method of quantification) is an acceptable practice to compensate for matrix effects. In this study, all analytes except cortisone exhibited weak matrix effects in both urine (−6.56 to 7.30%) and serum (−13.9 to 18.2%), indicating that the optimized method adequately removed interfering matrix components (Table 2 and Table 3). However, a moderate ion enhancement was found for cortisone in urine (29.6%) and serum (38.7%), which could be explained by the lack of isotope labelled cortisone available in this study (^13^C_3_-cortisol was used as the internal standard for cortisone). Use of isotopically labelled cortisone would reduce the uncertainty in measurement associated with matrix-effect for this analyte.

The sensitivity of the method was determined as LOD and LOQ in both human urine and serum. In human urine, the LODs ranged from 0.04 ng/mL (estradiol) to 0.28 ng/mL (11-dehydrocorticosterone), and the LOQs were between 0.14 ng/mL (estradiol) and 0.92 ng/mL (11-dehydrocorticosterone) (Table 2). In human serum, the LODs ranged from 0.11 ng/mL (estrone) to 0.35 ng/mL (pregnenolone), and the LOQs were between 0.38 ng/mL (estrone) and 1.18 ng/mL (pregnenolone) (Table 3). Thus, the method provides adequate sensitivity for the determination of 19 steroid hormones in human urine and serum/plasma. The method sensitivities were similar to those reported in the literature (e.g., LODs: 0.20–1.00 ng/mL in urine [20], and 0.04–1.35 in fetal bovine serum [5]) although the earlier studies used different methods to calculate LOD. In particular, the LODs for estrogens in urine in our study (0.04–0.17 ng/mL) were significantly lower than those reported in an earlier study (0.20–0.83 ng/mL) [20].

### 3.4. Method Application

**Figure 4:**
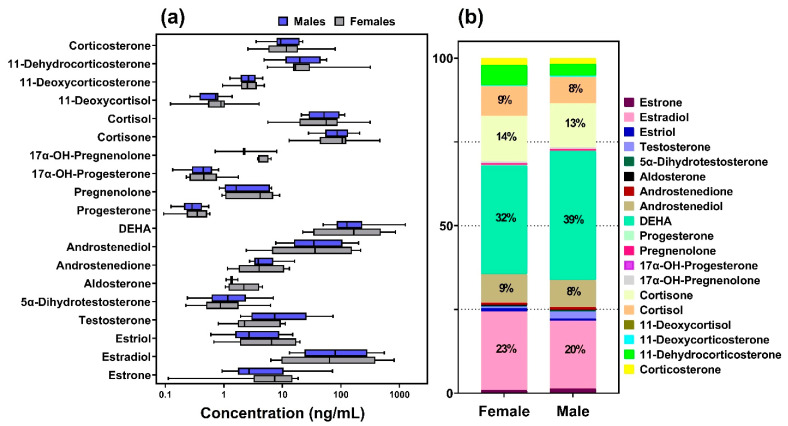
Concentrations (**a**) and relative distribution (**b**) of 19 steroid hormones measured in urine from 11 males and 9 females from New York, United States. Mean concentrations were used in the calculation of the percent contribution.

The validated method was applied for the analysis of 20 spot urine samples collected from healthy volunteers (11 males aged 18–64 y, and 9 females aged 15–59 y) during May–June 2022 from New York City. Sixteen analytes were found with detection frequencies (DFs) ≥ 80% in urine samples. The average concentrations of all analytes ranged from 0.34 to 284 ng/mL. In both males and females, DEHA was the most abundant (accounting for 39% and 32% of the total concentrations in males and females, respectively), followed by estradiol (20% and 23%, respectively), cortisol (13% and 14%, respectively), cortisone (8% and 9%, respectively), and androstenediol (8% and 9%, respectively) (Figure 4 and Table S4). Representative chromatograms of all analytes in urine samples are shown in Figure S3.

The method was applied in the analysis of human plasma by measuring all analytes in SRM1950. Among the 19 steroid hormones, 15 analytes were quantified at concentrations ranging from 0.14 (estrone) and 81.4 ng/mL (cortisol). Aldosterone, 11-deoxycorticosterone, estradiol, and estriol were below the respective LODs in the sample. In pooled human serum purchased from Sigma-Aldrich, 12 out of the 19 analytes were found at concentrations in the range of 0.23 (11-deoxycortisol) to 72.5 ng/mL (cortisol) (Table S5). Representative chromatograms of all analytes in serum samples are shown in Figure S4. Overall, the method is suitable for the determination of 19 steroid hormones in human urine and serum/plasma of the general population.

## 4. Conclusions

We developed and validated a HPLC-MS/MS method for simultaneous determination of 19 steroid hormones in human urine and serum/plasma. Liquid-liquid extraction with MTBE/EtAc (5:1, *v/v*) yielded acceptable recoveries of all analytes from these matrices. Chromatographic and mass spectrometric conditions were optimized to separate and detect the hormones at physiologically relevant concentrations. Dansylation of estrone, estradiol, and estriol improved their chromatographic resolution and sensitivities.

The method was validated for accuracy, precision, sensitivity, linearity, and through the analysis of certified plasma standard reference material. The method was applied in the analysis of 19 hormones in real human urine and serum/plasma. The method can also be used in the analysis of steroid hormones in other matrices (e.g., hair and saliva) with slight modifications. The method can be applied in the analysis of steroid hormones in human urine and serum/plasma in population-based biomonitoring studies.

## Figures and Tables

**Table 1:** Target analytes (19 steroid hormones), isotopically labeled internal standards, and their MRM parameters. MRM parameters include precursor ion (Q1), product ion (Q3), declustering potential (DP), entrance potential (EP), collision energy (CE), collision cell exit potential (CXP), and dwell time.

Name	CAS	Q1 *(m*/*z*)	Q3 (*m*/*z*)	DP (V)	CE (V)	EP (V)	CXP (V)	Dwell (ms)
**Target analytes**								
DC-Estrone		504	171	125	35	10	15	30
DC-Estradiol		506	171	125	35	10	15	30
DC-Estriol		522	171	125	35	10	15	30
Testosterone	58-22-0	289	97	100	29	10	12	30
5α-Dihydrotestosterone	521-18-6	291	255	100	22	10	14	30
Androstenedione	63-05-8	287	97	172	25	10	14	30
Androstenediol	521-17-5	273	255	93	17	10	8	30
DEHA	53-43-0	271	253	80	23	10	12	30
Progesterone	57-83-0	315	97	130	23	10	10	30
Pregnenolone	145-13-1	317	299	98	13	10	16	30
17α-OH-Progesterone	68-96-2	331	109	114	39	10	6	30
17α-OH-Pregnenolone	387-79-1	333	297	80	23	10	18	30
Cortisone	53-06-5	361	163	152	31	10	20	30
Cortisol	50-23-7	363	121	144	29	10	12	30
11-Deoxycortisol	152-58-9	347	97	161	27	10	10	30
11-Deoxycorticosterone	64-85-7	331	97	160	27	10	16	30
11-Dehydrocorticosterone	72-23-1	331	97	168	26	10	11	30
Corticosterone	50-22-6	347	329	100	23	10	18	30
Aldosterone	52-39-1	361	343	120	24	10	12	30
**Internal standards**								
DC-^13^C_3_-Estrone		507	171	125	35	10	15	30
DC-^13^C_2_-Estradiol		508	171	125	35	10	15	30
DC-^13^C_3_-Estriol		525	171	125	35	10	15	30
^13^C_3_-Testosterone		292	100	120	27	10	14	30
^13^C_3_-5α-Dihydrotestosterone		294	258	130	21	10	12	30
^13^C_3_-Dehydroepiandrosterone		274	256	125	14	10	14	30
^13^C_3_-Progesterone		318	100	170	30	10	6	30
^13^C_2_-D_2_-Pregnenolone		321	303	110	11	10	22	30
^13^C_3_-17α-OH-Progesterone		334	100	138	27	10	12	30
^13^C_2_-D_2_-17α-OH-Pregnenolone		319	301	90	15	10	16	30
^13^C_3_-Cortisol		366	124	152	30	10	22	30
^13^C_3_-11-Deoxycortisol		350	112	132	32	10	17	30
^13^C_3_-11-Deoxycorticosterone		334	100	154	27	10	18	30
D_4_-Corticosterone		351	333	126	17	10	18	30
D_4_-Aldosterone		365	347	100	26	10	24	30

Abbreviations: DC-Estrone, dansylated estrogen; DC-Estradiol, dansylated estradiol; DC-Estriol, dansylated estriol; DEHA, dehydroepiandrosterone.

**Table 2:** Method validation parameters for the analysis of 19 steroid hormones in human urine.

Analytes	R ^a^	LOD (ng/mL)	LOQ (ng/mL)	ME%	10 ng/mL			20 ng/mL			200 ng/mL		
					Recovery%	CV%		Recovery%	CV%		Recovery%	CV%	
						Intra-Day	Inter-Day		Intra-Day	Inter-Day		Intra-Day	Inter-Day
**Estrogen**													
Estrone	0.9990	0.17	0.56	−6.56	111 ± 9	8.39	3.24	114 ± 3	2.78	2.39	105 ± 5	4.61	0.55
Estradiol	0.9992	0.04	0.14	0.92	93.3 ± 11.5	12.4	2.84	120 ± 10	8.67	3.10	118 ± 11	9.05	4.08
Estriol	0.9981	0.13	0.42	−2.16	110 ± 5	4.18	9.68	109 ± 3	3.12	6.83	99.6 ± 5.3	5.31	3.65
**Androgen**													
Testosterone	0.9997	0.14	0.46	−0.81	113 ± 5	4.20	0.71	119 ± 1	0.60	1.34	115 ± 3	2.10	1.71
5α-Dihydrotestosterone	0.9974	0.15	0.49	−2.51	82.5 ± 1.3	1.50	4.17	85.0 ± 1.9	2.20	3.21	82.7 ± 2.0	2.40	0.74
Androstenedione	0.9994	0.22	0.73	7.30	109 ± 13	11.7	2.39	109 ± 11	10.4	2.17	119 ± 7	6.20	3.68
Androstenediol	0.9994	0.23	0.76	0.49	102 ± 12	11.7	2.56	119 ± 8	6.40	0.56	99.8 ± 4.8	4.83	0.65
DEHA	0.9996	0.24	0.81	−1.67	117 ± 6	4.95	3.88	91.7 ± 0	0.00	4.27	105 ± 8	7.45	2.58
**Progestogen**													
Progesterone	0.9990	0.15	0.50	−2.65	141 ± 11	7.50	3.93	132 ± 8	6.30	4.45	117 ± 8	6.60	4.77
Pregnenolone	0.9998	0.23	0.75	−2.48	106 ± 6	6.00	1.77	111 ± 1	0.90	2.70	110 ± 4	3.72	2.29
17α-OH-Progesterone	0.9997	0.21	0.70	0.75	110 ± 1	0.60	1.49	115 ± 13	11.2	2.31	113 ± 4	3.30	1.69
17α-OH-Pregnenolone	0.9998	0.12	0.41	−2.45	112 ± 4	3.10	3.86	112 ± 1	1.10	1.00	96.8 ± 4.0	4.20	2.43
**Corticosteroid**													
Cortisone	0.9985	0.13	0.44	29.6	101 ± 12	11.5	4.53	115 ± 8	7.08	3.41	117 ± 4	3.47	4.25
Cortisol	0.9998	0.20	0.65	−1.74	81.7 ± 15.5	19.0	1.34	100 ± 9	9.35	1.20	96.5 ± 4.3	4.49	3.12
11-Deoxycortisol	0.9995	0.19	0.64	5.84	112 ± 3	2.37	2.63	116 ± 4	3.80	4.37	106 ± 3	2.76	1.17
11-Deoxycorticosterone	0.9998	0.25	0.83	−2.47	97.1 ± 9.9	10.2	1.29	99.2 ± 1.4	1.45	2.42	99.9 ± 2.0	2.02	1.58
11-Dehydrocorticosterone	0.9990	0.28	0.92	5.41	86.7 ± 5.5	6.35	2.56	90.2 ± 3.2	3.56	1.82	84.1 ± 2.5	2.93	3.20
Corticosterone	0.9993	0.26	0.85	5.52	107 ± 4	3.37	2.20	101 ± 2	1.72	1.39	101 ± 1.3	1.31	1.13
Aldosterone	0.9997	0.19	0.63	4.42	95.5 ± 2.6	2.77	7.16	101 ± 10	9.47	7.59	90.0 ± 0.3	0.32	4.59

Abbreviation: DEHA, dehydroepiandrosterone; ME, matrix effect; LOD, limit of detection; LOQ, limit of quantification; CV, coefficient of variation. ^a^ R value in urine matrix.

**Table 3:** Method validation parameters for the analysis of 19 steroid hormones in human serum.

Analytes	R ^a^	LOD (ng/mL)	LOQ (ng/mL)	ME%	10 ng/mL			20 ng/mL			200 ng/mL		
					Recovery%	CV%		Recovery%	CV%		Recovery%	CV%	
						Intra-Day	Inter-Day		Intra-Day	Inter-Day		Intra-Day	Inter-Day
**Estrogen**													
Estrone	0.9998	0.11	0.38	1.09	112 ± 5	4.08	2.18	103 ± 3	2.97	6.06	97.3 ± 3.7	3.79	0.78
Estradiol	0.9989	0.14	0.48	0.92	88.5 ± 3.8	4.33	3.17	92.3 ± 8.9	9.63	1.14	93.3 ± 1.8	3.29	2.68
Estriol	0.9982	0.17	0.55	−6.49	110 ± 2	1.89	2.46	114 ± 5	3.97	2.39	99.0 ± 3.1	3.15	1.13
**Androgen**													
Testosterone	0.9958	0.22	0.75	−0.12	118 ± 8	6.37	2.16	116 ± 6	5.19	1.26	119 ± 1	0.64	2.63
5α-Dihydrotestosterone	0.9998	0.18	0.60	0.00	104 ± 7	6.28	4.78	114 ± 3	2.67	0.78	108 ± 1	0.80	3.02
Androstenedione	0.9985	0.16	0.52	18.2	114 ± 3	2.68	4.09	117 ± 3	2.58	1.48	110 ± 3	2.73	2.08
Androstenediol	0.9985	0.24	0.81	5.34	96.1 ± 1.2	1.20	3.88	104 ± 6	5.30	3.00	99.7 ± 1.4	1.45	2.20
DEHA	0.9990	0.17	0.56	−10.2	102 ± 5	5.05	6.43	102 ± 8	7.62	2.40	107 ± 3	2.84	3.89
**Progestogen**													
Progesterone	0.9997	0.17	0.58	−7.08	110 ± 5	4.48	4.40	115 ± 5	4.24	6.72	110 ± 3	2.62	5.82
Pregnenolone	0.9992	0.35	1.18	−0.62	96.6 ± 12.1	12.5	11.5	91.5 ± 1.3	1.45	8.55	100 ± 2	2.18	1.75
17α-OH-Progesterone	0.9994	0.13	0.42	2.99	109 ± 6	5.36	2.95	108 ± 2	1.84	1.42	100 ± 1	1.00	1.00
17α-OH-Pregnenolone	0.9974	0.24	0.80	−13.9	101 ± 3	3.04	5.80	100 ± 7	6.60	4.14	92.8 ± 3.5	3.73	2.51
**Corticosteroid**													
Cortisone	0.9990	0.25	0.84	38.7	119 ± 9	7.33	4.33	118 ± 8	6.58	1.04	119 ± 5	4.21	2.61
Cortisol	0.9981	0.24	0.79	−3.48	90.0 ± 14.1	15.7	1.24	80.0 ± 17.3	21.7	0.96	94.0 ± 8.8	9.32	2.06
11-Deoxycortisol	0.9997	0.17	0.58	7.22	107 ± 3	2.46	2.55	111 ± 2	1.88	1.99	105 ± 3	2.72	1.91
11-Deoxycorticosterone	0.9989	0.21	0.70	0.64	97.2 ± 1.6	1.68	1.01	103 ± 4	3.67	2.22	96.0 ± 3.5	3.61	2.04
11-Dehydrocorticosterone	0.9981	0.23	0.78	6.76	106 ± 9	8.04	7.61	107 ± 1	1.08	4.73	103 ± 2	2.03	4.22
Corticosterone	0.9995	0.17	0.56	−11.0	94.7 ± 6.4	6.71	0.16	100 ± 4	4.36	2.66	88.7 ± 1.3	1.49	1.83
Aldosterone	0.9997	0.22	0.73	9.73	102 ± 6	5.59	1.41	106 ± 5	4.34	4.74	98.8 ± 4.7	4.70	1.11

Abbreviation: DEHA, dehydroepiandrosterone; ME, matrix effect; LOD, limit of detection; LOQ, limit of quantification; CV, coefficient of variation. ^a^ R value in serum matrix.

**Table 4:** Steroid hormones measured in plasma Standard Reference Material (SRM1950) using the method developed in this study and compared to the certified reference values. Reference values were only available for cortisol, progesterone, and testosterone.

Analytes	Our Results (ng/mL)	Reference Value (ng/mL)	Variation%
**Estrogen**			
Estrone	0.14	n.a.	--
Estradiol	<LOD	n.a.	--
Estriol	<LOD	n.a.	--
**Androgen**			
Testosterone	2.47	2.214	11
5α-Dihydrotestosterone	0.20	n.a.	--
Androstenedione	0.74	n.a.	--
Androstenediol	0.58	n.a.	--
DEHA	1.99	n.a.	--
**Progestogen**			
Progesterone	1.36	1.482	8.6
Pregnenolone	0.41	n.a.	--
17α-OH-Progesterone	0.58	n.a.	--
17α-OH-Pregnenolone	0.94	n.a.	--
**Corticosteroid**			
Cortisol	81.4	83.9	3
Cortisone	20.9	n.a.	--
11-Deoxycortisol	0.17	n.a.	--
11-Deoxycorticosterone	<LOD	n.a.	--
11-Dehydrocorticosterone	0.67	n.a.	--
Corticosterone	1.57	n.a.	--
Aldosterone	<LOD	n.a.	--

Abbreviation: n.a., not available; DEHA, dehydroepiandrosterone; LOD, limit of detection.

## Data Availability

Not applicable.

## Supplementary Materials

The following supporting information can be downloaded at: https://www.mdpi.com/article/10.3390/toxics10110687/s1. Supplementary method: sample cleanup procedure using SPE. Figure S1. Representative LC-MS/MS chromatograms of 19 steroid hormones without derivatization. Concentrations of the target analytes were 100 ng/mL; Injection volume was 10 µL. Figure S2. Comparison of pregnenolone in serum following extraction with LLE, Bond Elut C18, Bond Elut Plexa, and Bond Elut NEXUS. Analyte concentration was 100 ng/mL, and the injection volume was 10 µL. Figure S3. Representative chromatograms of 19 steroid hormones found in human urine (injection volume: 10 µL). Figure S4. Representative chromatograms of 19 steroid hormones found in human serum (injection volume: 10 µL). Table S1. Optimized MRM parameters for the estrogens. Table S2. Comparison of the spike-recoveries of all analytes in serum following extraction with LLE, Bond Elut C18 (60 mg/3 mL), Bond Elut Plexa (60 mg/3 mL), and Bond Elut NEXUS (60 mg/3 mL). Analytes were spiked at 100 ng/mL, injection volume was 10 µL. Table S3. R value of the calibration curve of all analytes in neat solution. Table S4. Steroid hormone concentrations measured in twenty human urine samples from the general population in New York, USA, in 2022. Table S5. Steroid hormone concentrations measured in the pooled human serum purchased from Sigma-Aldrich.
