# Supplementary material for: Determination of 19 Steroid Hormones in Human Serum and Urine Using Liquid Chromatography-Tandem Mass Spectrometry
Source: Toxics. 2022 Nov 12;10(11):687. doi: 10.3390/toxics10110687 (PMC9699436; doi:10.3390/toxics10110687)
Supplement: Supplementary file 1 [file toxics-10-00687-s001.zip › toxics-1991351-supplementary.pdf]

Supporting Information

Determination of 19 Steroid Hormones in Human Serum and Urine Using Liquid Chromatography-Tandem Mass Spectrometry

Zhong-Min Li [1,2] and Kurunthachalam Kannan [1,2,*]

[1] Department of Pediatrics, New York University Grossman School of Medicine, New York, NY 10016, USA
[2] Department of Environmental Medicine, New York University Grossman School of Medicine, New York, NY 10016, USA
* Correspondence: kurunthachalam.kannan@nyulangone.org; Tel.: +1-(212)-263-1546

Submission to: Toxics

Sample cleanup procedure using SPE

A 500-μL aliquot of urine sample was pipetted into a 15-mL glass tube. Following spiking 2.5–12.5 ng of isotopically labeled internal standards, 500 μL of 1 M NH_4OAc buffer (pH 5.5) containing 5 mg/mL of L-ascorbic acid and 20 μL of ALS enzyme (2000 units) was added. After gentle mix, the samples were incubated overnight (~ 15 h) at 37 °C with shaking at 100 rpm (Jeio Tech Co., Seoul, Korea). After incubation, 1 mL of HPLC-grade water was added into each sample, and the samples were subjected for ultra-sonication for 15 min. The samples were then loaded onto SPE cartridges (Bond Elut C18, Bond Elut Plexa, and Bond Elut NEXUS) which have been preconditioned with 2 mL of MeOH and 2 mL of water. The cartridges were then washed with 2 mL of water:MeOH (95:5, *v*/*v*) and dried for 3 min using a vacuum pump. The analytes were eluted into a 15-mL glass tube using 3 mL MeOH. The elute was evaporated to dryness under gentle N_2 at 25 °C. Thereafter, 125 μL of sodium bicarbonate buffer (0.1 M, pH 9.0) and 125 μL of dansyl chloride (1 mg/mL in acetone) were added, vortexed vigorously (~30 sec), and immediately kept at 60 °C for 5 min. The samples were then transferred into amber glass vials for LC-MS/MS analysis.

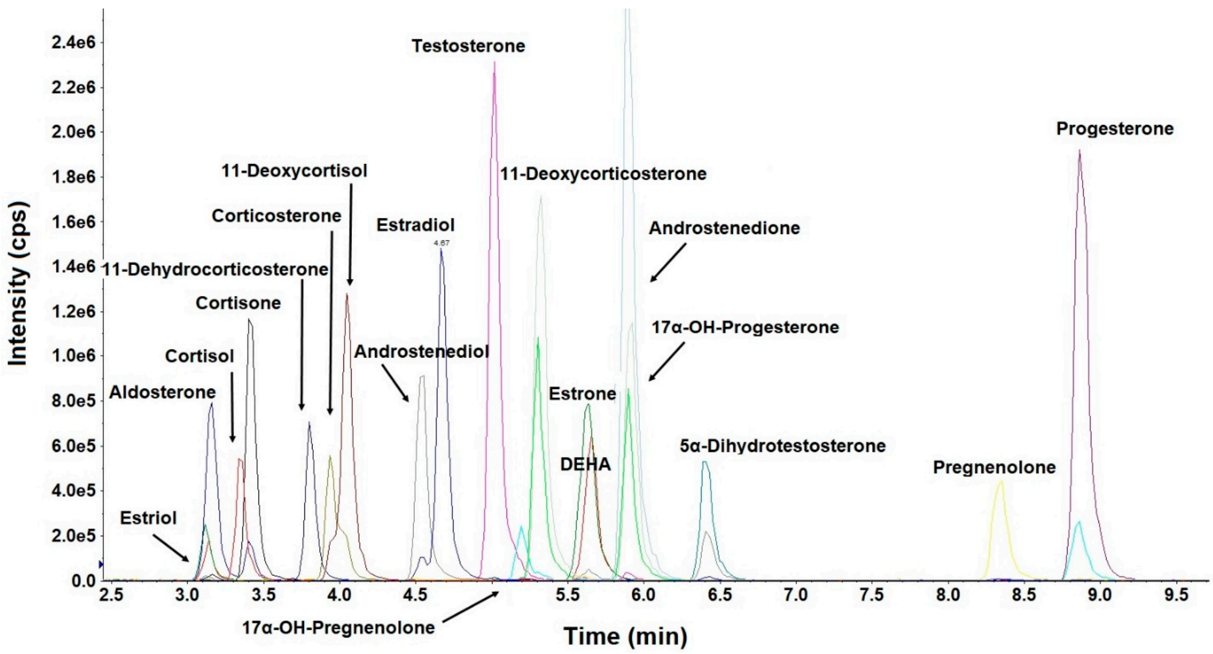

Figure S1 Representative LC-MS/MS chromatograms of 19 steroid hormones without derivatization. Concentrations of the target analytes were 100 ng/mL; Injection volume was 10 μL.

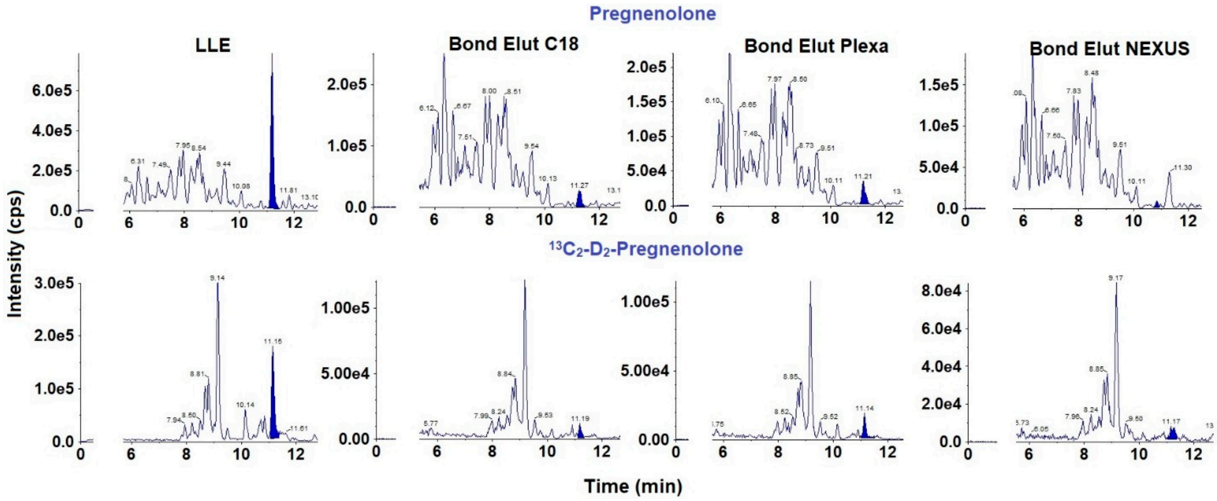

Figure S2 Comparison of pregnenolone in serum following extraction with LLE, Bond Elut C18, Bond Elut Plexa, and Bond Elut NEXUS. Analyte concentration was 100 ng/mL, and the injection volume was 10 μL.

Analytes	Internal standards
**Estrogen**	
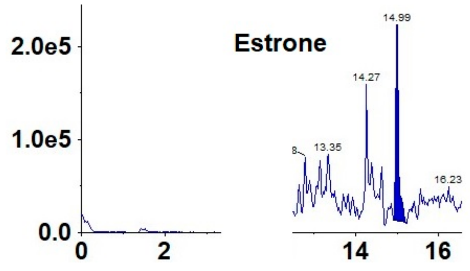	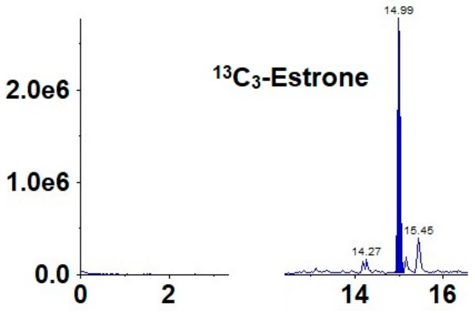
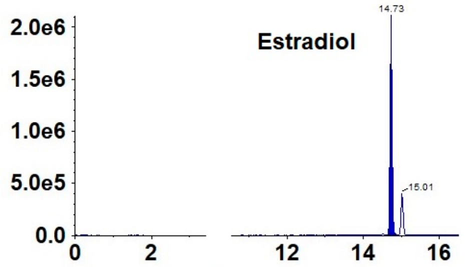	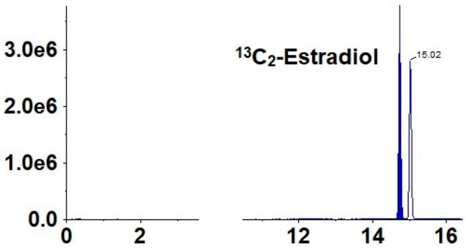
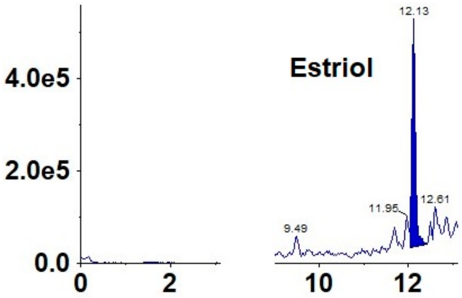	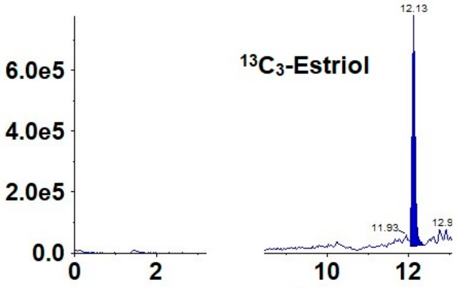
**Androgen**	
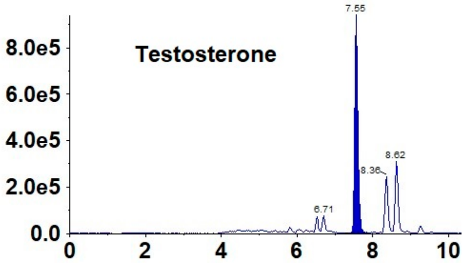	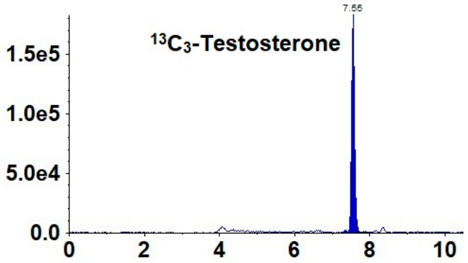
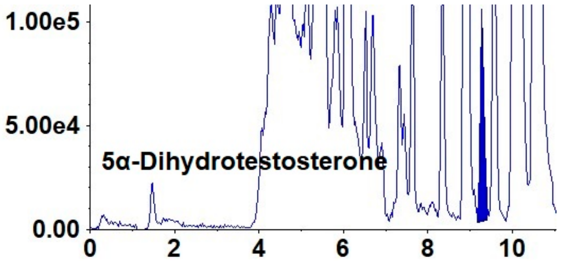	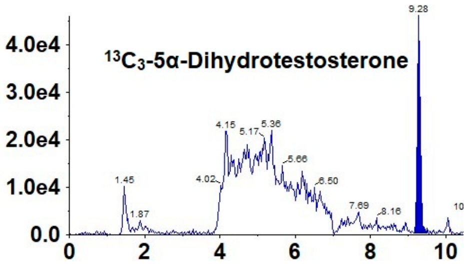

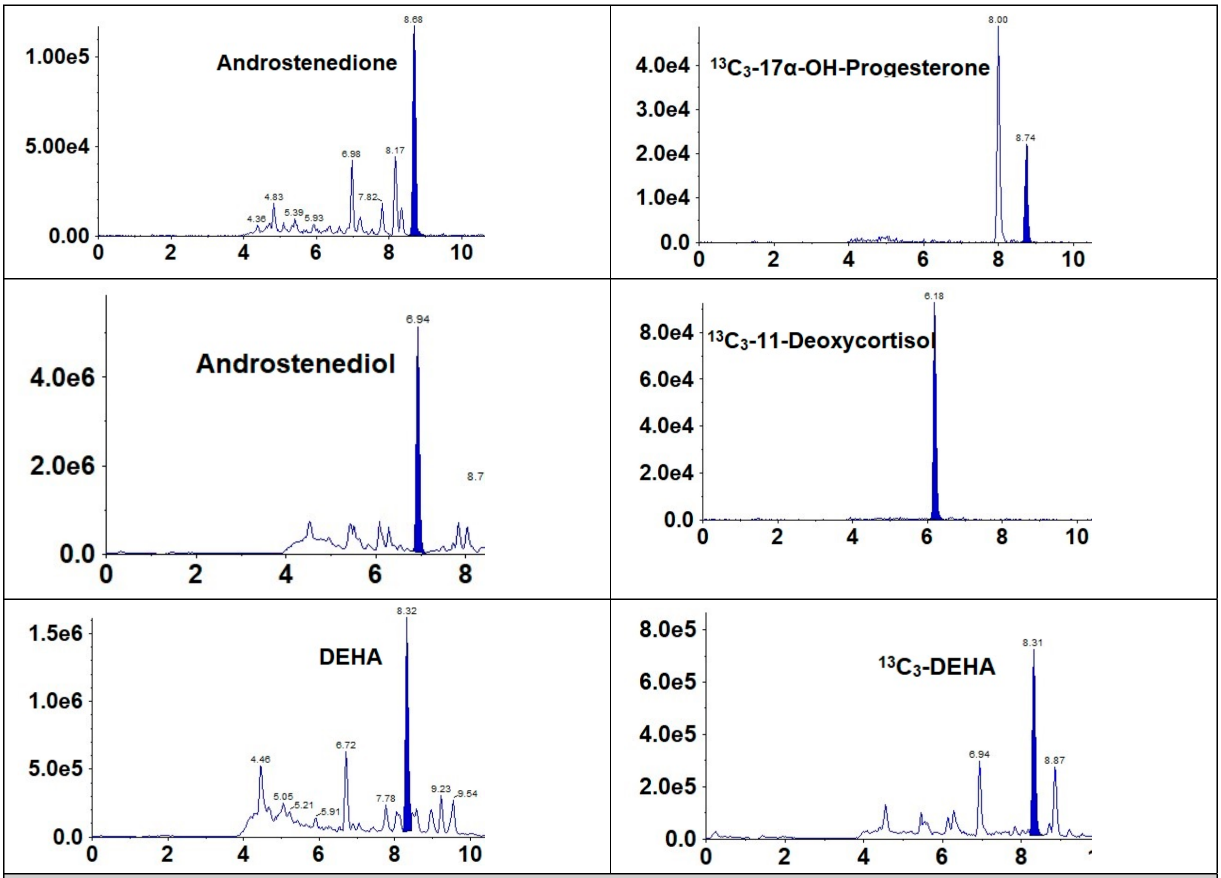

Progestogen

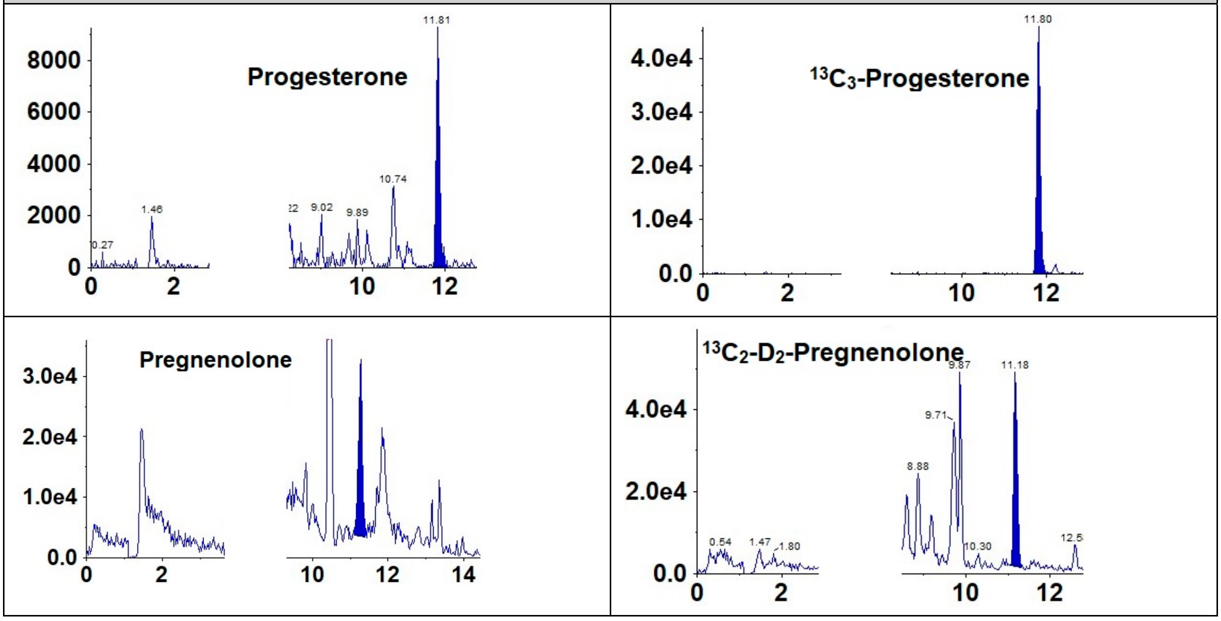

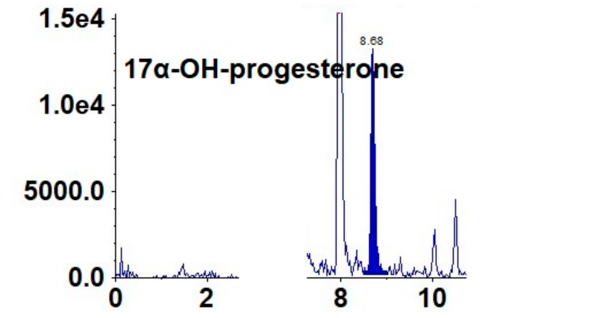

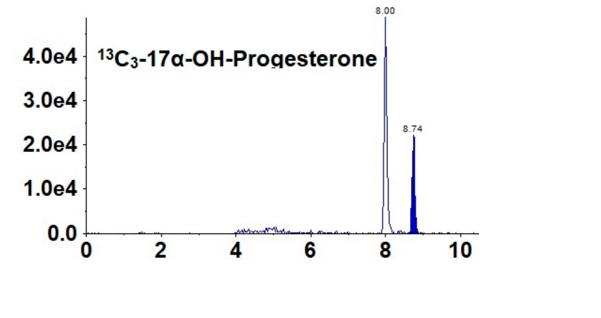

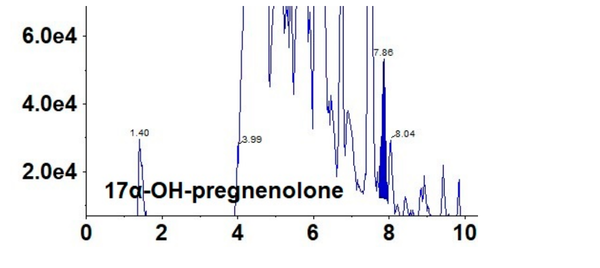

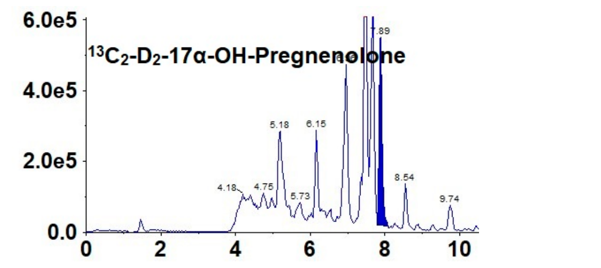

Corticosteroid

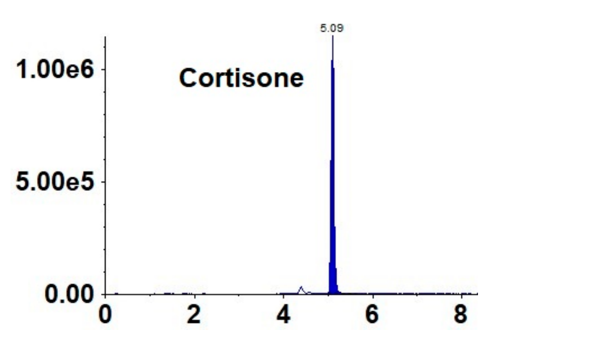

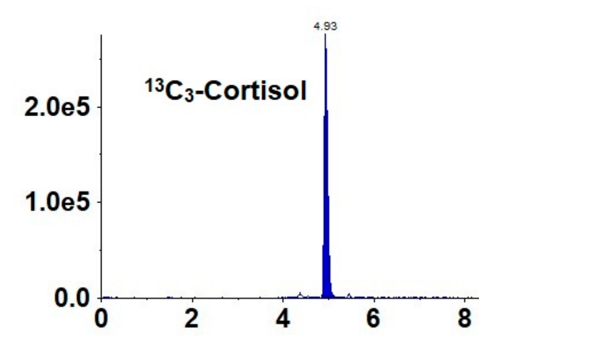

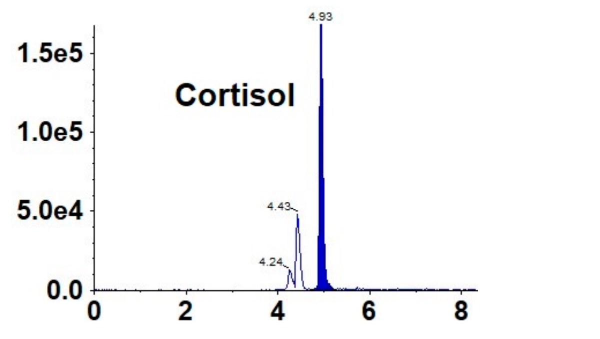

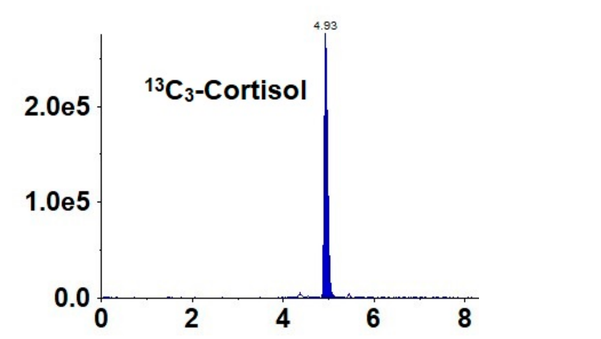

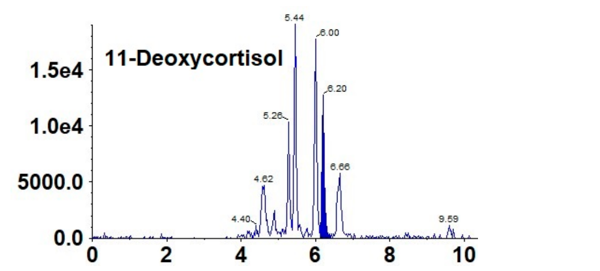

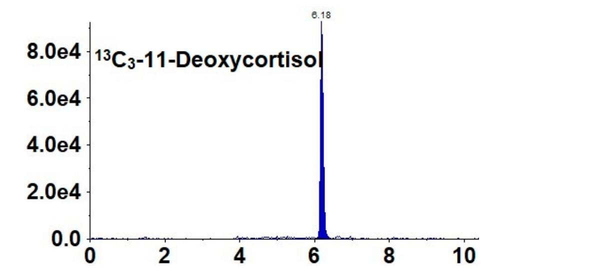

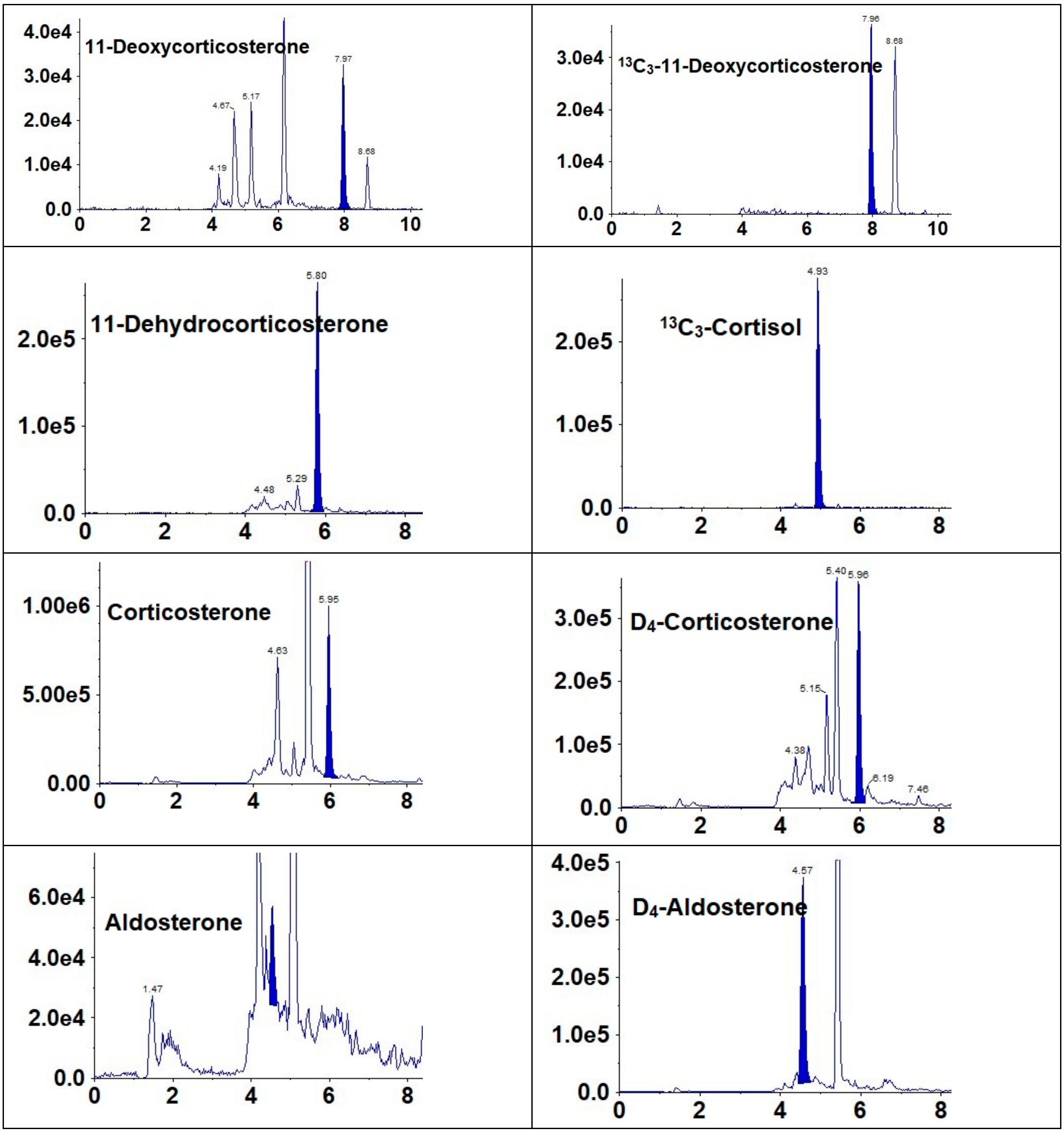

Figure S3 Representative chromatograms of 19 steroid hormones found in human urine (injection volume: 10 μL).

Analytes	Internal standards
**Estrogen**	
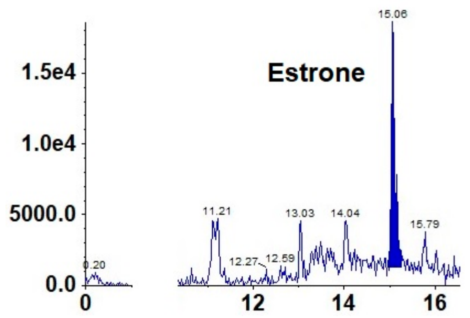	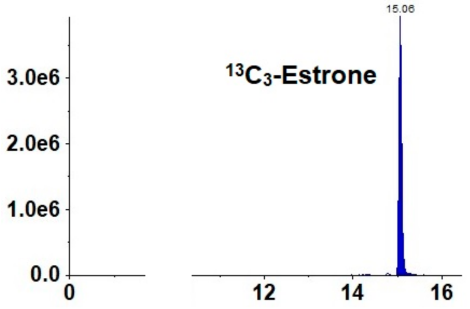
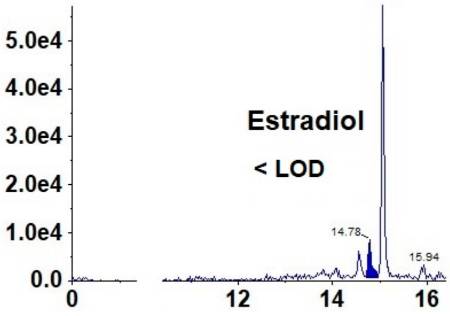	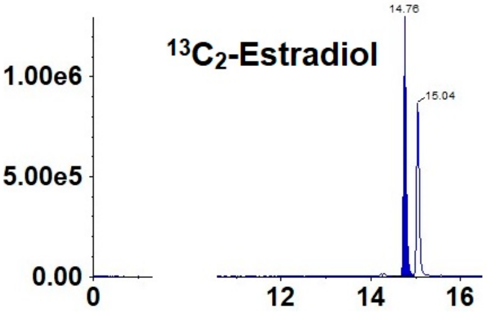
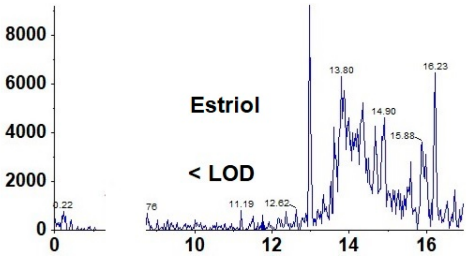	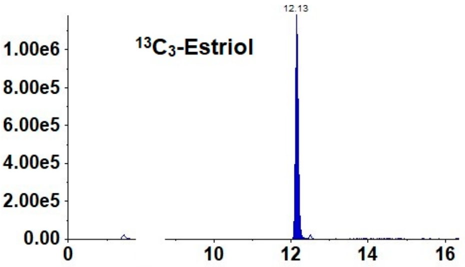
**Androgen**	
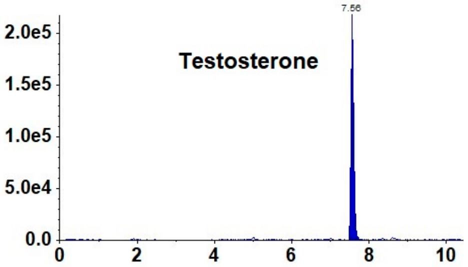	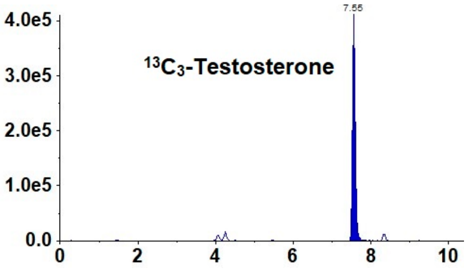
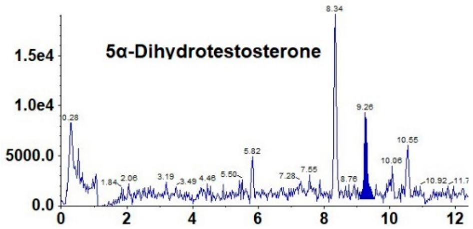	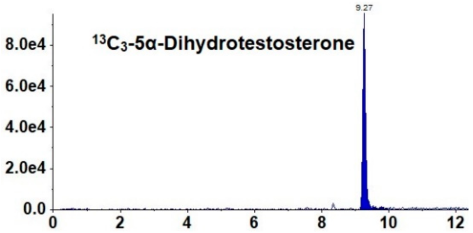

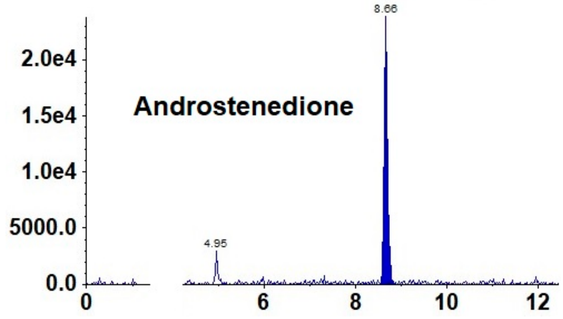

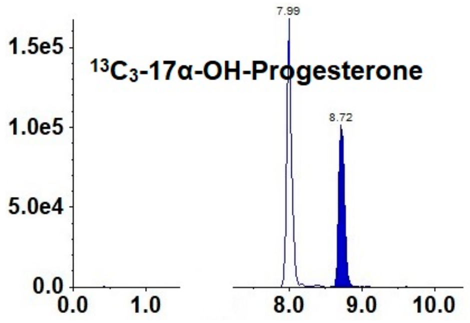

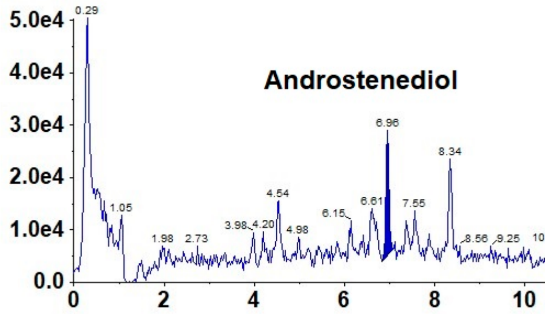

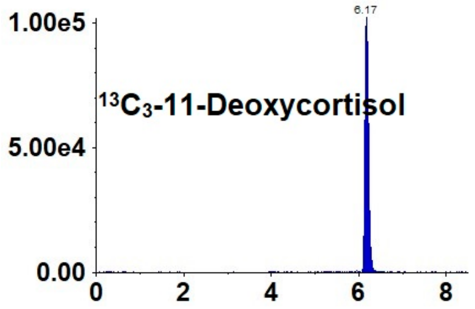

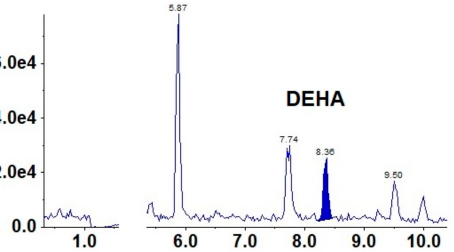

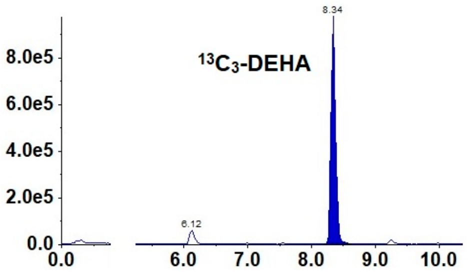

Progestogen

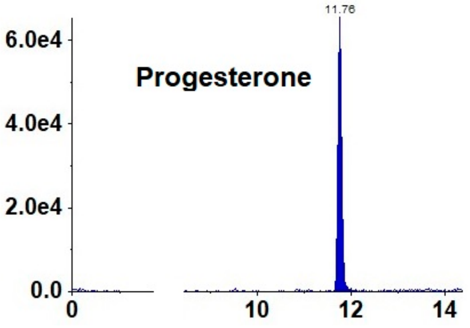

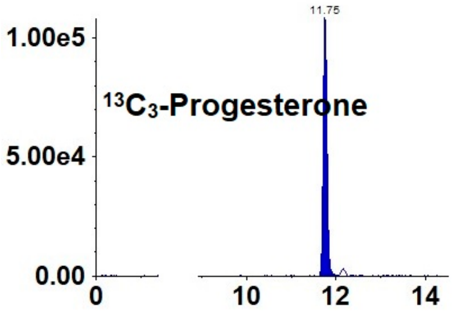

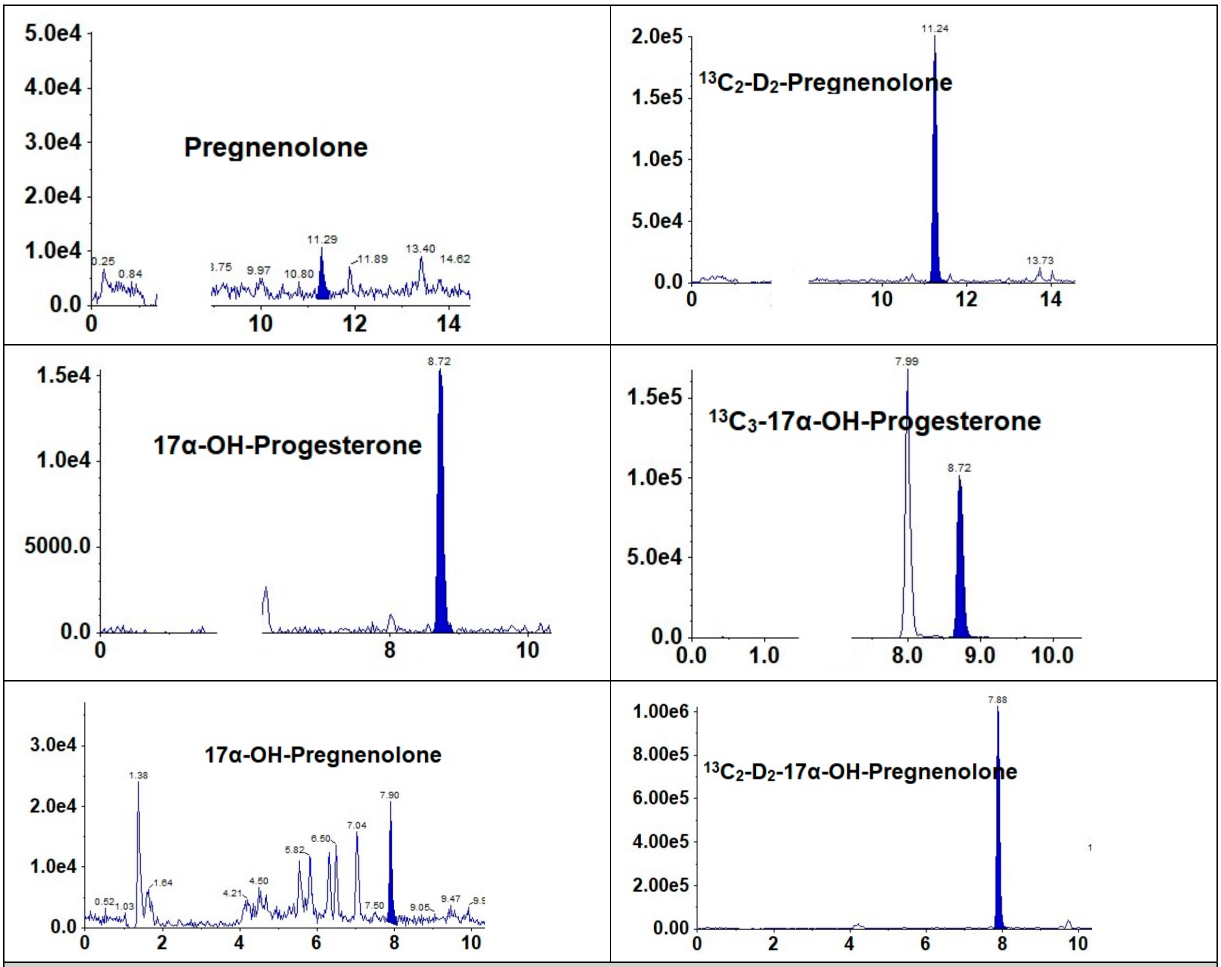

Corticosteroid

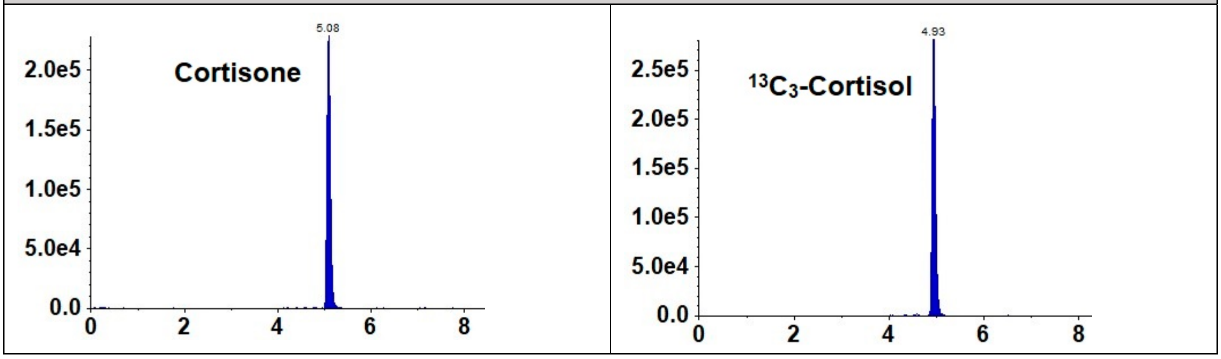

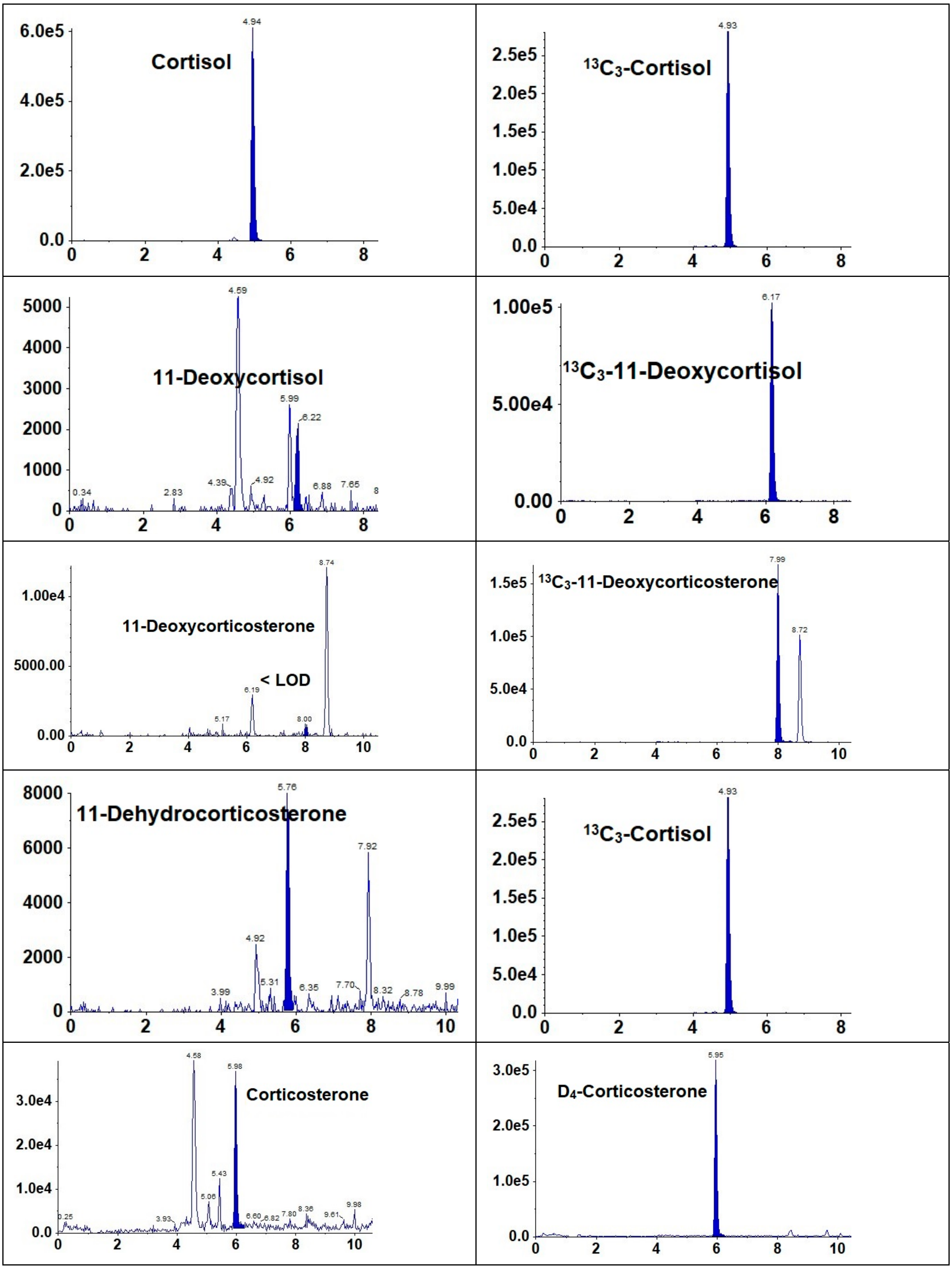
Cortisol
4.94
6.0e5
4.0e5
2.0e5
0.0
0 2 4 6 8
13C3-Cortisol
4.93
2.5e5
2.0e5
1.5e5
1.0e5
5.0e4
0.0
0 2 4 6 8
11-Deoxycortisol
4.59
5.99
6.22
0.34
2.83
4.39
4.92
6.88
7.65
5000
4000
3000
2000
1000
0
0 2 4 6 8
13C3-11-Deoxycortisol
6.17
1.00e5
5.00e4
0.00
0 2 4 6 8
11-Deoxycorticosterone
< LOD
8.74
6.19
5.17
8.00
1.00e4
5000.00
0.00
0 2 4 6 8 10
13C3-11-Deoxycorticosterone
7.99
8.72
1.5e5
1.0e5
5.0e4
0.0
0 2 4 6 8 10
11-Dehydrocorticosterone
5.76
7.92
4.92
3.99
5.31
6.35
7.70
8.32
8.78
9.99
8000
6000
4000
2000
0
0 2 4 6 8 10
13C3-Cortisol
4.93
2.5e5
2.0e5
1.5e5
1.0e5
5.0e4
0.0
0 2 4 6 8
Corticosterone
4.58
5.98
5.43
5.06
0.25
3.93
6.60
6.82
7.80
8.36
9.61
9.98
3.0e4
2.0e4
1.0e4
0.0
0 2 4 6 8 10
D4-Corticosterone
5.95
3.0e5
2.0e5
1.0e5
0.0
0 2 4 6 8 10

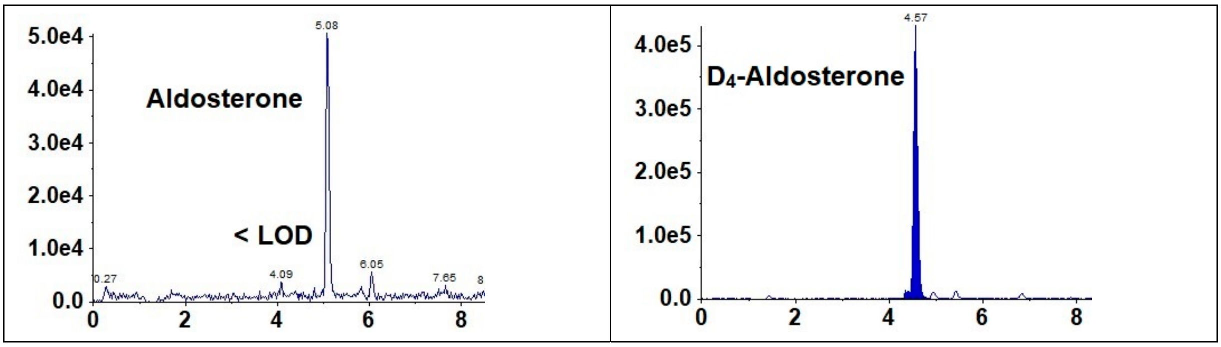

Figure S4 Representative chromatograms of 19 steroid hormones found in human serum (injection volume: 10 μL)

Table S1 Optimized MRM parameters for the estrogens.

Name	CAS	Q1 (*m*/*z*)	Q3 (*m*/*z*)	DP (V)	CE (V)	EP (V)	CXP (V)	Dwell (ms)
**Target analytes**								
Estrone	53-16-7	271	159 (q)	110	28	10	19	20
			133 (c)	138	29	10	12	20
Estradiol	50-28-2	255	159 (q)	123	23	10	10	20
			133 (c)	146	22	10	10	20
Estriol	50-27-1	271	253 (q)	74	13	10	29	20
			159 (c)	113	35	10	14	20
**Internal standards**								
$^{13}C_3$-Estrone		274	256	120	16	10	15	20
$^{13}C_2$-Estradiol		257	161	126	26	10	10	20
$^{13}C_3$-Estriol		274	256	100	14	10	16	20

Abbreviations: q, quantification transition; c, confirmation transition.

Table S2 Comparison of the spike-recoveries of all analytes in serum following extraction with LLE, Bond Elut C18 (60 mg/3 mL), Bond Elut Plexa (60 mg/3 mL), and Bond Elut NEXUS (60 mg/3 mL). Analytes were spiked at 100 ng/mL, injection volume was 10 µL.

Analytes	LLE (%)	Bond Elut C18 (%)	Bond Elut Plexa (%)	Bond Elut NEXUS (%)
**Estrogen**				
Estrone	99.3 ± 2.5	88.0 ± 3.3	92.6 ± 2.3	90.5 ± 7.0
Estradiol	86.5 ± 1.4	64.4 ± 3.5	61.7 ± 1.5	61.6 ± 3.6
Estriol	99.1 ± 5.5	93.0 ± 2.1	88.2 ± 1.5	90.7 ± 2.5
**Androgen**				
Testosterone	130 ± 4	119 ± 1	126 ± 4	125 ± 4
5α-Dihydrotestosterone	104 ± 3	85.2 ± 1.6	92.7 ± 1.7	98.9 ± 6.9
Androstenedione	110 ± 3	128 ± 9	135 ± 4	127 ± 6
Androstenediol	97.2 ± 2.4	93.6 ± 4.6	96.1 ± 4.1	103 ± 4
DEHA	107 ± 6	109 ± 3	109 ± 2	105 ± 7
**Progestogen**				
Progesterone	103 ± 3	103 ± 3	99.0 ± 4.2	98.1 ± 1.5
Pregnenolone	90.5 ± 3.6	---[a]	58.2 ± 8.6	---[a]
17α-OH-Progesterone	103 ± 7	96.0 ± 5.6	95.2 ± 4.3	92.1 ± 2.8
17α-OH-Pregnenolone	93.1 ± 6.5	91.7 ± 2.6	98.5 ± 3.4	98.1 ± 2.0
**Corticosteroid**				
Cortisone	129 ± 3	146 ± 9	146 ± 10	141 ± 7
Cortisol	93.0 ± 1.0	95.0 ± 7.8	96.7 ± 8.7	84.8 ± 2.5
11-Deoxycortisol	105 ± 2	96.8 ± 2.3	95.7 ± 6.0	98.0 ± 5.3
11-Deoxycorticosterone	93.8 ± 5.7	85.6 ± 0.8	87.6 ± 3.6	88.8 ± 3.2
11-Dehydrocorticosterone	101 ± 5	114 ± 5	115 ± 4	110 ± 6
Corticosterone	88.2 ± 0.5	73.0 ± 2.2	76.6 ± 1.8	70.6 ± 3.3
Aldosterone	97.5 ± 3.2	87.7 ± 2.9	92.1 ± 2.3	96.2 ± 2.1

[a]recoveries were not calculated.

Table S3 R value of the calibration curve of all analytes in neat solution.

Analytes	R
**Estrogen**	
Estrone	0.9997
Estradiol	0.9985
Estriol	0.9980
**Androgen**	
Testosterone	0.9998
5α-Dihydrotestosterone	0.9994
Androstenedione	0.9993
Androstenediol	0.9999
DEHA	0.9998
**Progestogen**	
Progesterone	0.9995
Pregnenolone	0.9997
17α-OH-Progesterone	0.9994
17α-OH-Pregnenolone	0.9994
**Corticosteroid**	
Cortisone	0.9973
Cortisol	0.9997
11-Deoxycortisol	0.9995
11-Deoxycorticosterone	0.9999
11-Dehydrocorticosterone	0.9995
Corticosterone	0.9997
Aldosterone	0.9995

Table S4 Steroid hormone concentrations measured in twenty human urine samples from the general populations in New York, USA in 2022.

Anlaytes	DF%	Min (ng/mL)	Max (ng/mL)	Mean (ng/mL)	SD (ng/mL)	GM (ng/mL)
**Estrogen**						
Estrone	100	0.11	72.0	9.43	15.7	4.20
Estradiol	100	6.35	810	175	229	70.3
Estriol	85	< LOD	19.9	6.65	6.53	3.95
**Androgen**						
Testosterone	100	0.81	73.5	10.8	16.9	5.36
5α-Dihydrotestosterone	95	< LOD	7.00	1.92	2.13	1.18
Androstenedione	100	1.15	16.2	5.83	4.09	4.64
Androstenediol	100	2.41	218	66.6	71.1	32.9
DEHA	100	22.3	1265	284	333	152
**Progestogen**						
Progesterone	100	0.09	0.58	0.34	0.15	0.30
Pregnenolone	65	< LOD	8.95	3.71	2.83	2.66
17α-OH-Progesterone	95	< LOD	1.76	0.51	0.37	0.42
17α-OH-Pregnenolone	35	< LOD	8.00	4.16	2.44	3.37
**Corticosteroid**						
Cortisone	100	13.1	462	107	97.0	80.7
Cortisol	100	5.65	316	65.9	67.7	45.3
11-Deoxycortisol	100	0.12	4.00	0.87	0.79	0.69
11-Deoxycorticosterone	100	0.95	4.92	2.84	1.08	2.63
11-Dehydrocorticosterone	100	4.85	317	37.4	67.3	21.4
Corticosterone	100	2.57	79.5	15.3	16.4	11.0
Aldosterone	45	< LOD	4.58	2.14	1.28	1.87

Abbreviations: DF, detection frequency; SD, standard deviation; GM, geometric mean; DEHA, dehydroepiandrosterone.

Table S5 Steroid hormone concentrations measured in the pooled human serum purchased from Sigma-Aldrich.

Analytes	Mean (ng/mL)	SD (ng/mL)
**Estrogen**		
Estrone	< LOD	
Estradiol	< LOD	
Estriol	< LOD	
**Androgen**		
Testosterone	4.70	0.03
5α-Dihydrotestosterone	0.30	0.01
Androstenedione	0.52	0.05
Androstenediol	0.63	0.03
DEHA	9.05	0.51
**Progestogen**		
Progesterone	< LOD	
Pregnenolone	< LOD	
17α-OH-Progesterone	0.55	0.02
17α-OH-Pregnenolone	0.74	0.16
**Corticosteroid**		
Cortisone	12.7	0.35
Cortisol	72.5	2.29
11-Deoxycortisol	0.23	0.01
Aldosterone	< LOD	
11-Deoxycorticosterone	< LOD	
11-Dehydrocorticosterone	0.63	0.08
Corticosterone	1.35	0.10
